# Avocado Consumption Increases Macular Pigment Density in Older Adults: A Randomized, Controlled Trial

**DOI:** 10.3390/nu9090919

**Published:** 2017-08-23

**Authors:** Tammy M. Scott, Helen M. Rasmussen, Oliver Chen, Elizabeth J. Johnson

**Affiliations:** Jean Mayer USDA Human Nutrition Research Center on Aging, Tufts University, Boston, MA 02111, USA; tammy.scott@tufts.edu (T.M.S.); helen.rasmussen@tufts.edu (H.M.R.); oliver.chen@tufts.edu (O.C.)

**Keywords:** lutein, avocado, macular pigment, bioavailability, cognition, older adult

## Abstract

Lutein is selectively incorporated into the macula and brain. Lutein levels in the macula (macular pigment; MP) and the brain are related to better cognition. MP density (MPD) is a biomarker of brain lutein. Avocados are a bioavailable source of lutein. This study tests the effects of the intake of avocado on cognition. This was a six-month, randomized, controlled trial. Healthy subjects consumed one avocado (*n* = 20, 0.5 mg/day lutein, AV) vs. one potato or one cup of chickpeas (*n* = 20, 0 mg/day lutein, C). Serum lutein, MPD, and cognition were assessed at zero, three, and six months. Primary analyses were conducted according to intent-to-treat principles, with repeated-measures analysis. At six months, AV increased serum lutein levels by 25% from baseline (*p* = 0.001). C increased by 15% (*p* = 0.030). At six months, there was an increase in MPD from baseline in AV (*p* = 0.001) and no increase in C. For both groups, there was an improvement in memory and spatial working memory (*p* = 0.001; *p* = 0.032, respectively). For AV only there was improved sustained attention (*p* = 0.033), and the MPD increase was related to improved working memory and efficiency in approaching a problem (*p* = 0.036). Dietary recommendations including avocados may be an effective strategy for cognitive health.

## 1. Introduction

Lutein and zeaxanthin are dietary carotenoids that cross the blood brain barrier and exclusively accumulate in the macular region of the retina. In the macula, lutein and zeaxanthin are referred to as macular pigment (MP) [1]. MP is believed to have a role in visual function and health by acting as a blue light filter and also possibly as an antioxidant and anti-inflammatory agent [2]. Lutein is transported in the circulation primarily on high density lipoproteins (HDL) [3], and HDL levels have been reported to be significantly related to MP optical density (MPD) [4]. HDL has been reported to be a specific transporter of lutein into neural tissue [5]. Thus increasing the dietary intake of lutein, as well as altering the lipoprotein profile, may impact MPD.

MPD has been reported to be significantly related to cognitive function in both young and older adults [6,7,8]. Studies in nonhuman primates and humans show that retinal lutein is significantly associated with lutein brain concentrations [9,10]. Thus MPD is a non-invasive biomarker for brain concentrations of lutein. Lutein concentrations in the older adult brain are positively associated with a variety of pre-mortem cognitive measures [11]. Furthermore, lutein supplementation significantly improves verbal fluency scores in healthy older adults [12]. The effect of lutein-containing foods on cognitive function is not known. Lutein containing foods include green leafy vegetables, squash, broccoli, and eggs [13]. Avocados are a particularly bioavailable source [14]. An avocado-enriched diet improves lipoprotein profiles, with increases in HDL and decreases in low-density lipoproteins (LDL) [15,16]. Based on the sum of these findings, the primary objective of this study was to test the effects of the intake of avocado versus potatoes or chickpeas on cognition in older adults. A secondary objective was to investigate the effect of avocado consumption on anti-oxidation, anti-inflammation, and the lipoprotein profile.

## 2. Materials and Methods

### 2.1. Subjects

Forty-eight healthy, non-smoking women and men were recruited from the general population from January 2013 through January 2014. All subjects completed the study by July 2014. The subjects underwent a screening examination that included a medical history, physical examination, and a routine blood clinical chemistry profile. The inclusion criteria included low intakes of lutein-rich foods (less than three servings/week of green leafy vegetables, broccoli, eggs). The exclusion criteria included any history of liver, kidney or pancreatic disease; anemia; active bowel disease or resection; bleeding disorders; and avocado, potato or chickpea allergies. Moreover, individuals taking medications interfering with fat absorption (e.g., bile acid sequestrants, exetimibe) were excluded. Other exclusion criteria included the current use of steroids, or antipsychotic, antimanic, anti-inflammatory, monoamine inhibitors, or dementia medications. Additionally, the use of any nutrient supplement for the previous two months was grounds for exclusion. Aspirin and non steroidal anti-inflammatory drugs were not considered as grounds for exclusion.

### 2.2. Experimental Design

The study was designed as a randomized, controlled trial. The study protocol was approved by the Human Investigative Review Committee of Tufts University. Informed consent was obtained from all subjects. This study was registered at ClinicalTrials.gov (https://clinicaltrials.gov/ct2/show/NCT01620567?term=avocado&rank=4; Clinical Trial Registry number: NCT01621646).

### 2.3. Study Protocol

Upon enrollment, the research dietitian (HMR) randomized subjects to either group using a randomization plan generator (http://www.randomization.com/). The randomization assignment was known to the Metabolic Kitchen Laboratory staff only. At baseline and Months three and six, the subjects visited the Jean Mayer U.S. Department of Agriculture Human Nutrition Center on Aging at Tufts University and provided a fasting blood sample. Serum were separated from red blood cells (800 g, 10 min) and were stored at −70 °C until analyzed. Measures of cognitive function and MPD were performed at these visits. A food frequency questionnaire was also given [17] and analyzed by the research dietitian. Dietary intake data were analyzed using Nutrition Data System for Research software (version 2016, University of Minnesota, Minneapolis, MN, USA), developed by the Nutrition Coordinating Center (NCC), University of Minnesota, Minneapolis, MN, USA. The subjects were instructed to not change their dietary habits throughout the study period.

The subjects visited the Nutrition Center monthly to pick up supplies of fresh avocados (Hass Avocado™, Mission Viejo, CA, USA, approximately 135 g/day of avocado containing 0.5 mg lutein, 13 g/day monounsaturated fats) or potatoes (Costa Produce™, Boston, MA, USA)/chickpeas (Progresso™, Minneapolis, MN, USA) (containing no lutein, <1 g/day monounsaturated fats). The lutein content of the avocados was measured by our laboratory using previously published methods [18] and was found to be 0.40 ± 0.01 mg/100 g (edible portion). The monounsaturated fat content of avocados is 9.8 g/100 g [19]. Foods in each group were selected to be isocaloric. The subjects had the option to have avocados shipped weekly directly to their homes. The subjects were also offered pre-made foods containing avocados, potatoes, or chickpeas (soup, pesto, sauce, dip, ice cream) for easy incorporation into their daily meals. However, only a small proportion of the foods (<10%) were consumed in these forms. The subjects were instructed to consume one avocado/day (treatment group) or one potato or one cup of chickpeas (control group) or the equivalent contained in pre-made foods. The total amount of lutein provided by the avocados over the study period was 90 mg. At the monthly visits, compliance was monitored by an interview conducted by a dietitian with specific questions on the subject’s daily intake of the intervention foods and a review of compliance calendars. The maintenance of dietary habits was also determined at these times. Semi-monthly telephone monitoring occurred to check on subjects’ food supplies and to query them about any situation that may have occurred with food products. The subjects were encouraged to contact the kitchen laboratory or the dietitian if there was any question about the quality or amount of food supplies they received.

### 2.4. Serum Analysis for Carotenoids and Biomarkers of Oxidative Stress and Inflammation

Serum lutein was extracted and analyzed by high performance liquid chromatography by using a method previously described [20]. The oxidative status was measured using low density lipoprotein (LDL) resistance against oxidation [21]. β-Amyloid and C-reactive protein were measured in serum as markers of inflammation using a direct solid-phase enzyme immunoassay (Innogenetics N.V., Ghent, Belgium) and a chemiluminescent assay (Diagnostic Products Corporation, Los Angeles, CA, USA), respectively.

### 2.5. Lipoprotein Analysis

Protocol was as specified in a Beckman Coulter AU400 standard operating procedure insert for total cholesterol, HDL, LDL, and triglycerides. (Beckman Coulter Inc., Brea, CA, USA).

### 2.6. Measurement of Macular Pigment Density

A psychophysical measurement of MPD, using heterochromatic flicker photometry, was used as a measure of lutein and zeaxanthin in neural tissue [22]. The measurements were conducted using a modified macular densitometer (Macular Metrics Corp, Rehoboth, MA, USA), which was simplified in its construction from the original densitometer described earlier [23] and modified for clinical use. MPD at 0.5° eccentricity and at two wavelengths (460 and 500 nm) was assessed. The standardized protocol for measuring MPD by heterochromatic flicker photometry has been shown to be reliable and safe in older adults [24].

### 2.7. Cognitive Tests

The subjects underwent cognitive testing using a computerized battery of cognitive tests designed to evaluate several cognitive domains, including memory, processing speed, and attention [25] (Table 1, CANTAB, Cambridge Cognition Ltd., Cambridge, UK). These tests required a finger-operated subject response on a touch-screen tablet personal computer. A set of scripted instructions was provided for each test.

### 2.8. Sample Size Calculation

Standard sample size calculation procedures estimate that 20/group gives an 80% chance that a 0.05 level test of significance will find a significant difference in MPD. This is based on the study by Wenzel et al., who reported a significant increase of 0.09 OD in MPD with one egg/day (containing lutein/zeaxanthin at 0.33 mg/day) for 12 weeks [26].

### 2.9. Statistical Analyses

The treatment groups were compared for possible differences in characteristics and outcome measures at baseline using independent samples, *t*-Test, or Chi-Square where appropriate. The primary analyses were conducted according to intent-to-treat principles. A repeated-measures analysis was utilized, with the dependent variable being changed to the follow-up time points. The independent variables were treatment, time (visit), and the treatment × time interaction. Type I error was controlled using a gatekeeping strategy; a hierarchical structure was identified as the treatment × time interaction, followed by the main effects, followed by specific within- and between- group comparisons. Pearson correlations were conducted to evaluate the relationships between measures. Comparisons were performed only at α = 0.05 if significance was obtained at α = 0.05 at the higher level in the hierarchy. The data are presented as mean ± SD. All significance testing was two-tailed using an alpha of 0.05. The data were analyzed with SPSS (version 22, Armonk, New York, NY, USA).

## 3. Results

### 3.1. Subject Characteristics

Forty subjects completed the study (83% of enrolled). Eight subjects (five avocado, three control) dropped out of the study for the following reasons: Aversion to the study protocol (*n* = 2), diagnosis of colitis (*n* = 1), did not follow study directions (*n* = 2), and did not show for study visit and could not be reached (*n* = 3). No adverse events were reported.

There were no significant differences between the two groups in baseline measures of age, body mass index (kg/m^2^), education, lutein/zeaxanthin, monounsaturated fat intake, (Table 2), serum concentrations of lutein and zeaxanthin, C-reactive protein, β-amyloid, blood lipids, LDL oxidation, or MPD (Table 3). 

Among the cognitive measures, there were also no significant differences in baseline measures of cognitive function with the exception of a delayed matching sample, a measure of short term visual memory for which the avocado group had a higher (better) measure. (Table 4).

Compliance with consumption of the food interventions (days consumed/total days of study) as assessed by an interview with a dietitian compliance calendars was 98% and 99% for the avocado group and control group, respectively. There was no significant change in dietary habits or body weight for either group throughout the study.

### 3.2. Serum Analysis for Carotenoids and Biomarkers of Oxidative Stress and Inflammation

There was no treatment by time interaction or main effect of treatment for either lutein or zeaxanthin. There was a main effect for time for serum lutein and zeaxanthin. In post-hoc analyses of the avocado group, there was a significant increase in serum lutein concentrations from baseline at both three and six months, with increases that were >25% for both time points. There were no changes in serum zeaxanthin throughout the study period in the avocado group (Table 3). In the control group, there was a 15% increase in serum lutein at six months that was significantly greater than baseline (Table 3). Serum zeaxanthin concentrations in the control group were significantly increased from baseline by >20% at both time points.

There were no changes in measures of oxidative stress and inflammation throughout the study in either group.

### 3.3. Lipoproteins

There was no treatment by time interaction or main effect of treatment or time for HDL, LDL, or total cholesterol (Table 3). There was a trend for a treatment by time interaction for triglycerides (*p* = 0.061). At six months, there was a trend for the triglyercides to decrease from baseline in the avocado group (*p* = 0.075) and increase in the control group (*p* = 0.06). The change (six months baseline) in HDL was correlated with the change in both serum lutein and zeaxanthin (*r* = 0.43 and 0.54; *p* = 0.058 and 0.014, respectively) in the avocado group only, and the change in triglycerides was negatively correlated with the change in serum zeaxanthin in the control group (*r* = −0.41, *p* = 0.073).

### 3.4. Macular Pigment Density

There was no treatment by time interaction or main effect of treatment for MPD. There was a main effect of time. In post hoc analyses, MPD increased from baseline by more than 25% at both three and six months in the avocado group (Table 3). These changes were not related to the changes in serum concentrations of lutein or zeaxanthin. In the control group, MPD significantly increased from baseline by approximately 17% in the control group at three months (Table 3) and was significantly related to the change in serum zeaxanthin (*r* = 0.607, *p* = 0.005) but not to the change in serum lutein. The increase in MPD or its relationship with serum zeaxanthin was not sustained at six months.

### 3.5. Cognitive Measures

There was no treatment by time interaction or main effect for treatment for any of the cognitive tests. There was a main effect for time for Spatial Working Memory, Rapid Visual Information Processing, Stockings of Cambridge, and Paired Associate Learning Test (Table 4). For the Paired Associate Learning test, Months 3 and 6 were significantly different than baseline for both groups (Table 4). In the avocado group, there was a significant improvement from baseline in the Spatial Working Memory measure at three months, as well as in the Stockings of Cambridge test at six months (Table 4). There were no other significant changes in cognitive measures for the control group.

### 3.6. Relationship between MPD and Cognitive Measures

In the avocado group, the change in MPD was significantly related to changes in Spatial Working Memory (*r* = 0.46, *p* = 0.041) and the efficiency in approaching a problem (*r* = 0.47, *p* = 0.036). There were no relationships between the change in MPD and the change in cognition in the control group.

## 4. Discussion

A dietary intervention with avocados was found to improve cognitive function. This improvement could be related to the increase in MPD, a biomarker of lutein contained in brain tissue [9,10]. The proposed mechanisms by which lutein benefits cognitive function in the elderly may involve its role as an antioxidant or anti-inflammatory agent [2]. However, in this study, no changes in oxidative stress or inflammation biomarkers were detected in either group. These measures were within a normal range at the start of the study, and therefore an improved antioxidant for anti-inflammatory status would have been difficult to detect. Other proposed mechanisms by which lutein is embedded in neural tissue include the modulation of functional properties of synaptic membranes, along with certain changes in the physiochemical and structural features of these membranes [27].

It is interesting to note that the control group had a significant increase in one of the cognitive measures with time (PAL). We speculate that this may be attributed to a learning effect, demonstrating the importance of including a control group in intervention trials. Furthermore, it is probable that phytonutrients other than lutein in chickpeas and potatoes may improve cognitive function.

Our study finds that avocados have a positive impact on lutein status, particularly in neural tissue. This finding confirms what other investigators have reported [14]. The high bioavailability of lutein contained in avocados has been previously reported [14]. Presumably adding oil, grinding, or cooking would further increase this bioavailability. It was not possible to test this in our study, given the low number of subjects who chose the option of prepared foods. Although the amount of lutein contained in avocados is relatively small (0.5 mg/medium avocado), this dose was extremely effective in increasing serum lutein. For comparison, we have reported that lutein supplementation (12 mg/day for 4 months) increased serum lutein by 0.22 nmol/L per mg lutein [28]. This present study finds an increase in serum of 0.93 nmol/L per mg lutein contained in avocado. The control group also had a small but significant increase in serum lutein at six months. This unexpected result may have been due to unaccounted for dietary changes during the study, although this was not indicated in the dietary interviews.

The daily consumption of avocados resulted in increases in serum lutein that were significantly related to increases HDL, the major transporter of lutein in the circulation [3]. Monounsaturated fatty acids from avocados have been reported to increase HDL in mild hypercholesterolemic patients [16]. Avocado consumption has also been reported to lower LDL-cholesterol and triglycerides [16,29]. Although the changes in lipoproteins were small but trending towards significance, simultaneous increases in serum lutein and HDL concentrations and decreases in LDL and triglycerides are likely to be effective in increasing lutein in neural tissue through an enrichment of lutein in HDL. Indeed, this research found that avocado consumption was particularly effective in increasing MPD. Higher lutein status, particularly as measured by MPD, is believed to be important for visual function and health [2]. We and others have reported on the significant relationships between MPD and cognitive function [6,7,8]. This relationship is likely due to MPD being a biomarker of lutein concentrations in brain tissue [9,10]. Among the carotenoids, lutein is preferentially taken up into brain tissue, and lutein concentrations are the most consistently related to cognitive function [11].

In our previous study of similar design and methods, we reported that a 12 mg/day lutein (+0.5 mg/day zeaxanthin) supplement for four months in older adults significantly increased MPD by 0.041 OD [29]. In this present study, the consumption of 0.5 mg/day lutein contained in avocado for six months significantly increased MP density by 0.101 OD. The total lutein/zeaxanthin doses given over the study period were 1440 mg and 90 mg for the lutein supplement and avocado, respectively. Thus, the avocado intervention increased MPD by more than double that of the supplement, with only a small fraction of the amount of lutein. This suggests that other components in avocado are particularly effective in the enrichment of neural lutein. The most likely components are monounsaturated fatty acids.

Compared to the avocado group, the control group had smaller but significant increases in MPD at three months. However, this was not sustained at the end of the study. This change was not related to cognitive function. This is likely because the change was small and transient.

One limitation to this study is the lack of a comparison of avocados with more commonly consumed sources of lutein, e.g., green leafy vegetables. However, despite the relatively smaller levels of lutein contained in avocados (0.4 mg/100 g), compared to spinach (13 mg/100 g), [13] significant increases in lutein status resulted from the daily consumption of avocados. We speculate that the lipid nature of avocados facilitates lutein bioavailability, similar to our report on the higher bioavailability of lutein contained in egg compared to that contained in spinach [30]. Another limitation is whether it is realistic to consume one avocado a day. Avocados may be perceived as high in fat. However, there were no significant weight gains in our study. This may be due to the satiety effect of avocados [31]. In our study, avocados were consumed fresh or incorporated into foods that could be eaten as a part of a meal (soup, pesto, sauce, dip, ice cream). This likely aided the high compliance and weight maintenance and illustrates that such dietary modification can be practical. However, the Dietary Guidelines recommend choosing a variety of fruits and vegetables [32]. Therefore, investigations on the effects of other lutein-rich foods on cognitive function are warranted.

## 5. Conclusions

This study is an example of how practical dietary choices can be of benefit to healthy aging. A dietary intervention with avocados was particularly effective in increasing MPD levels, a biomarker of brain lutein. Increases in MPD were related to better cognitive performance. Therefore, avocados could be an effective dietary strategy for cognitive health in the aging population.

## Figures and Tables

**Table 1:** Cognitive Test Battery [25].

Cognitive Test	Description	Outcome Measures
Attention Tests		
Test		Score
Choice Reaction Time (CRT)	Two-choice reaction time test with two possible stimuli and two possible responses	Mean correct latency (response speed)
Rapid Visual Information Processing (RVIP)	Test of sustained attention	A’—Signal detection measure of sensitivity to the target B”—Signal detection measure of the strength of trace required to elicit a response
Visual Memory Tests		
Test		Score
Delayed Match to Sample (DMS)	Test of simultaneous and delayed matching to sample	Percent correct (all delays)
Paired Associates Learning (PAL)	Assesses visual memory and new learning	Total errors (adjusted) Stages completed
Executive Function, Working Memory, and Planning Tests		
Test		Score
Spatial Span (SSP)	Assesses working memory	Span length
Spatial Span Reverse (SSP-R)	Assesses working memory	Span length
Spatial Working Memory (SWM)	Tests ability to retain spatial information and manipulate items in working memory	Between errors strategy
Stockings of Cambridge (SOC)	Test of spatial planning and spatial working memory	Problems solved in minimum moves; Mean total thinking time: four moves

**Table 2:** The baseline characteristics of healthy subjects consuming one medium avocado a day (Avocado, *n* = 20) or one medium potato or one cup of chickpeas a day (Control, *n* = 20) for six months.

	Avocado	Control
Age, years, mean (SD)	63.3 (11.1)	62.5 (9.2)
Body mass index, kg/m^2^, mean (SD)	24.1 (3.1)	24.2 (2.4)
Education (%), some college or greater	85	75
Sex (female/male)	14/6	11/9
Dietary lutein/zeaxanthin, mg/day, mean (SD)	3.0 (3.1)	2.8 (2.7)
Dietary protein, g/day (SD)	75.3 (41.9)	68.2 (44.0)
Dietary fat, g/day (SD)	74.3 (35.0)	66.2 (41.4)
Dietary carbohydrate, g/day (SD)	246.0 (130.5)	228.3 (130.8)
Dietary monounsaturated fatty acids, g/day (SD)	27.4 (12.8)	22.3 (11.5)

**Table 3:** Serum lutein, zeaxanthin, macular pigment density (MPD), blood lipids, markers of inflamation, and oxidative stress in healthy adults (>50 years) consuming one medium avocado a day (Avocado, *n* = 20) or one medium potato or one cup of chickpeas a day (Control, *n* = 20) for six months. Mean (SD).

	Avocado			Control		
	Baseline	3 Months	6 Months	Baseline	3 Months	6 Months ^a^
Lutein (nmol/L)	330 (139)	423 (111) ^b^	414 (147) ^b^	322 (134)	352 (153)	371 (168) ^c^
Zeaxanthin (nmol/L)	63 (17)	69 (45)	57 (30)	65 (33)	83 (47) ^d^	78 (40) ^e^
Macular pigment denisty (MPD), OD	0.393 (0.142)	0.484 (0.178) ^f^	0.494 (0.137) ^b^	0.380 (0.173)	0.445 (0.177) ^g^	0.424 (0.149)
Serum cholesterol (mg/dL)	219 (52)	224 (56)	209 (43)	200 (46)	209 (42)	205 (54)
high density lipoproteins (HDL) (mg/dL)	57 (21)	61 (23)	61 (22)	58 (16)	56 (14)	57 (18)
low-density lipoproteins (LDL) (mg/dL)	129 (43)	135 (37)	131 (25)	132 (37)	137 (37)	137 (45)
Triglycerides (mg/dL)	86 (64)	99 (82)	79 (57)	84 (55)	94 (84)	93 (58)
C-reactive protein, mg/L	2.0 (2.8)	2.0 (2.8)	1.7 (1.8)	1.3 (1.0)	2.3 (2.4)	1.6 (1.6)
β-Amyloid, pg/mL	7.7 (2.8)	7.9 (2.7)	7.5 (2.7)	9.6 (5.0)	9.2 (5.2)	9.7 (5.2)
LDL Oxidative lagtime (min)	88.2 (22.9)	91.2 (14.2)	89.1 (17.3)	84.5 (26.6)	86.2 (16.9)	85.0 (18.7)

^a^ *n* = 19 for blood measures due to loss of sample due to technical error. For each group, significance from baseline (*p* value): ^b^ 0.001; ^c^ 0.03; ^d^ 0.01; ^e^ 0.004; ^f^ 0.005; ^g^ 0.04; OD: optical density; HDL: high density lipoprotein; LDL: low density lipoprotein.

**Table 4:** Cognitive scores in healthy adults (>50 years) at baseline (0 month) and after three and six months of consuming one medium avocado a day (Avocado, *n* = 20) or one medium potato or one cup of chickpeas a day (Control, *n* = 20) for six months. Mean (SD).

	Avocado			Control		
	Baseline	3 Months	6 Months	Baseline	3 Months	6 Months
CRT *mean latency msec*	347.4 (55.4)	355.0 (53.2)	342.8 (56.8)	356.0 (70.6)	367.0 (76.5)	359.1 (75.5)
RVIP *signal detection* ^4^	0.86 (0.21)	0.92 (0.04)	0.93 (0.04)	0.90 (0.05)	0.92 (0.05)	0.92 (0.05)
DMS *percent correct all delays*	84.7 (12.8) ^a^	86.0 (10.4)	84.7 (13.5)	80.0 (9.4)	85.3 (11.2)	83.0 (12.1)
PAL *total errors (adjusted)* ^2^	28.0 (17.8)	17.5 (14.2) ^c^	19.5 (15.5) ^d^	27.3 (18.7)	17.9 (17.0) ^e^	16.8 (14.9) ^e^
SS Forward *highest span*	5.8 (1.2)	6.2 (1.3)	5.8 (1.2)	5.8 (1.1)	6.1 (1.3)	6.1 (1.5)
SS Reverse *highest span* ^1^	5.3 (1.6)	5.3 (1.3)	5.5 (1.4)	5.0 (1.0)	5.6 (1.4)	5.7 (2.0) ^b^
SWM errors ^3^	52.5 (19.6)	43.6 (19.8) ^f^	48.4 (17.3)	50.7 (19.1)	47.80 (17.6)	42.1 (19.8)
SOC *# completed in minimum moves* ^5^	7.8 (2.1)	8.7 (2.3)	8.8 (2.2)^c^	8.0 (2.0)	8.4 (2.2)	9.0 (2.7)

CRT: Choice Reaction Time; RVIP: Rapid Visual Information Processing (higher scores are better); DMS: Delayed Matching to Sample; PAL: Paired Associates Learning; SS: Spatial Span; SWM: Spatial Working Memory (higher scores are worse); SOC: Stockings of Cambridge. ^1^ significant time effect, no group or time × group interaction (*p* = 0.045, univariate and adjusted for age and education) ; ^2^univariate: significant time effect (*p* = 0.001); adjusted with no main effects or interactions (although Months 3 and 6 are significantly different than baseline for both groups); ^3^ univariate: significant time effect (*p* = 0.032); adjusted for significant time effect, no group or time × group interactions; ^4^ univariate: significant time effect (*p* < 0.033) for the avocado group, no group or time × group interaction; adjusted for no main effects or interactions; ^5^ univariate: significant time effect (*p* = 0.029) for the avocado group, no group or time × group interaction; adjusted for no main effects or interactions. ^a^ Significantly different than the control at baseline (*p* = 0.045). For each group, signicantly different than baseline (*p* value): ^b^ 0.028; ^c^ 0.002; ^d^ 0.018; ^e^ 0.001; ^f^ 0.020; ^g^ 0.042.
